# A prospective life cycle assessment of global ammonia decarbonisation scenarios

**DOI:** 10.1016/j.heliyon.2024.e27547

**Published:** 2024-03-07

**Authors:** Johanna Boyce, Romain Sacchi, Earl Goetheer, Bernhard Steubing

**Affiliations:** a Institute of Environmental Sciences (CML), Leiden University, Einsteinweg 55, 2333 CC, Leiden, the Netherlands; b Laboratory for Energy Systems Analysis, Paul Scherrer Institute, Forschungsstrasse 111, 5232, Villigen, Switzerland; c Process and Energy, Faculty of Mechanical, Maritime and Materials Engineering, Technical University of Delft, Mekelweg 2, 2628 CD, Delft, the Netherlands

**Keywords:** Prospective LCA, Ammonia, Scenarios, IEA roadmap, IMAGE, Fertilizer

## Abstract

A prospective life cycle assessment was performed for global ammonia production across 26 regions from 2020 to 2050. The analysis was based on the IEA Ammonia Roadmap and IMAGE electricity scenarios model for three climate scenarios related to a mean surface temperature increase of 3.5 °C, 2.0 °C, and 1.5 °C by 2100. Combining these models with a global perspective and new life cycle inventories improves ammonia's robustness, quality, and applicability in prospective life cycle assessments. It reveals that complete decarbonisation of the ammonia industry by 2050 is unlikely from a life cycle perspective because of residual emissions in the supply chain, even in the most ambitious scenario. However, strong policies in the 1.5 °C scenario could significantly reduce climate impacts by up to 70% per kilogram of ammonia. The cumulative greenhouse gas emissions from the ammonia supply chain between 2020 and 2050 are estimated at 24, 21, and 15 gigatonnes CO_2_-equivalent for the 3.5 °C, 2.0 °C, and 1.5 °C scenarios, respectively. The paper examines challenges in achieving these scenarios, noting that electrolysis-based (yellow) ammonia, contingent on electricity decarbonisation, offers a cleaner production pathway. However, achieving significant GHG reductions is complex, requiring advancements in technologies with lower readiness, like carbon capture and storage and methane pyrolysis. The study also discusses limitations such as the need to reduce urea demand, potential growth in ammonia as a fuel, reliance on CO_2_ transport and storage, expansion of renewable energy, raw material scarcity, and the longevity of existing plants. It highlights potential shifts in environmental impacts, such as increased land, metal, and mineral use in scenarios with growing renewable electricity and bioenergy with carbon capture and storage.

## 1 Introduction

Understanding major industries' current and (potential) future environmental impacts is a complex task. Life cycle assessment (LCA) is a valuable tool to quantify the impacts of a product or process’ entire life cycle. However, LCA relies on the quality and relevance of the underlying data, making it less suitable for projections into a rapidly changing future. Prospective life cycle assessment (pLCA) is a tool used to address this limitation, as it assesses both direct and indirect environmental impacts of future product systems based on detailed scenarios of the development of technologies [[1], [2], [3]].

To avoid a temporal mismatch between foreground and background data, prospective life cycle inventory (pLCI) databases have been developed to represent future developments, mainly focusing on the power sector [4], with other sectoral integration efforts focusing on steel and cement production. However, the chemical industry, particularly its most prominent contributor, ammonia, responsible for almost half of its direct greenhouse gas (GHG) emissions in 2020 [5], has yet to undergo a detailed prospective life cycle assessment. This study pioneers a comprehensive global pLCA of the ammonia industry, a sector previously not studied in such detailed future-oriented environmental impact analysis.

Decarbonising heavy and hard-to-electrify industries, such as ammonia production, is a significant challenge that 194 countries have pledged to solve to align with the Paris Agreement objectives [[6], [7], [8]]. Ammonia and its derivatives are widely used as fertilisers, supporting the feeding of a significant portion of the global population [9].

The Haber-Bosch process, which uses nitrogen and hydrogen, is the primary method for producing ammonia [10]. The hydrogen comes from fossil fuel sources, particularly from steam reforming of natural gas (SMR), while the nitrogen is taken directly from the air. Nowadays, SMR supplies hydrogen for more than 70% of ammonia, while most of the remainder is made with hydrogen from coal gasification (which represents 85% of Chinese ammonia production) [7]. In both cases, CO_2_ is produced and either emitted or used on-site for urea synthesis by reaction with ammonia [11]. Captured CO_2_ can also be stored or used for other industrial processes or the food and beverage sectors [7]. Being tied to the food industry, environmental impacts associated with ammonia production correlate with population and economic growth [7]. A recent IEA roadmap described how the ammonia sector could combat this and align with climate goals [7].

Integrated assessment models (IAMs), such as IMAGE [12], produce scenarios for energy-intensive sectors that satisfy future socio-economic needs and respect given levels of radiative forcing of the atmosphere until 2100. Yet, the level of detail for the ammonia industry in IAMs is low. Furthermore, these models only quantify GHG emissions directly caused by the production process, leaving out 1) decarbonisation efforts in the upstream supply chain and the effects on downstream products and 2) other environmental impacts.

This study presents an approach that integrates the insights of scenarios for power and ammonia production into a prospective LCA framework. Adopting a life cycle perspective is crucial in evaluating the environmental impacts of ammonia production, remarkably, as IEA scenarios suggest a move towards decarbonisation with hydrogen and other technologies. While IAM and IEA scenarios focus mainly on direct emissions, an LCA approach captures other often-overlooked upstream impacts. This shift in impact, from direct emissions to upstream processes such as hydrogen production and raw material processing, is a vital aspect yet to be fully addressed. By including direct and indirect emissions, LCA provides a comprehensive view of ammonia production's environmental footprint, encompassing emissions from production and associated upstream activities. Further, this shift in impact can also operate from one type of environmental impact to another. This holistic understanding, made possible by combining these scenarios with LCA, is essential for accurately assessing the true environmental implications of greener ammonia production technologies.

Hence, integrating power and ammonia scenarios into LCA offers unprecedented insight into ammonia production's future environmental impacts. Doing so allows answering the following questions: How will the scale and technology mix of ammonia production for existing applications develop in the future? To what extent can the sector decarbonise when considering upstream supply chain emissions? And are there any environmental trade-offs to consider?

Additionally, the life cycle inventories and the integration approach developed in this study can be reused by LCA practitioners when ammonia products play an essential role in the system under study, improving the quality and robustness of future pLCAs.

Section 2 (Method) presents the approach to integrating sectoral projections into the LCA model, providing the reader with context surrounding ammonia production and its developments. Section 3 (Results and Interpretation) displays the life cycle GHG emissions for ammonia production for different regions and climate scenarios and highlights potential trade-offs with other environmental impacts. Finally, the effect of decarbonisation on downstream ammonia-consuming products is analysed. The findings are discussed further in Section 4 (Discussion). Section 5 (Conclusions and Recommendations) summarises the key takeaways.

## 2 Method

**Fig. 1:**
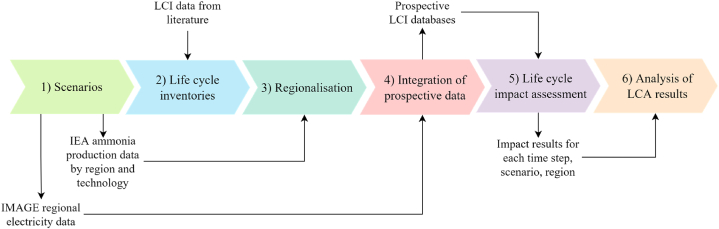
Method overview.

Six main steps, illustrated in Fig. 1, compose the approach. First, IEA scenarios for ammonia production are paired with scenarios from the IAM model IMAGE, which provides projections for electricity production. Next, life cycle inventories (LCI) are modelled based on literature (Step 2) and adjusted to match the different regions covered by the scenarios (Step 3). Fourth, the scenarios and the modelled LCI datasets are implemented in the LCA database ecoinvent. The fifth step involves producing LCA results for ammonia per kilogram across production routes, time, and scenarios. The projected production volumes also allow for calculating regional and global environmental impacts. In Step 6, LCA results are analysed.

### 2.1 Scenarios (Step 1)

IAMs produce scenarios for industrial sectors and future socio-economic needs based on shared socio-economic pathways (SSPs) and corresponding levels of atmospheric radiative forcing until 2100 with a representative concentration pathway (RCP). These explorative scenarios provide a consistent basis for producing pLCI databases [4,13,14]. Unfortunately, IAMs currently model ammonia production as a single process (SMR) without improvements or shifts to alternative production methods, meaning that the GHG emissions need to be compensated by other sectors. Hence, using the narratives provided by IAMs to explore the future production routes of ammonia production would not provide any valuable insights.

Based on a review of the literature and available scenarios, the IEA Ammonia Technology Roadmap [7], also referred to as the “IEA Roadmap” in this paper, is identified as a thorough illustration of potential futures for ammonia production. It includes regional and global production volumes and technology shares. It is selected as the basis of the ammonia scenarios in this study. The IEA Roadmap provides ammonia production projections for the Stated Policies Scenario (STEPS) and Sustainable Development Scenario (SDS) [7].

The IEA scenarios are therefore used with the electricity scenarios from the IMAGE IAM, which provide a model of the electricity sector for each world region and time step. For this exercise, the pathway SSP2, also known as *Middle of the road*, is chosen, along with three different RCPs, namely 6.0 (absence of mitigation measures), 2.6, and 1.9 W/m^2^, respectively, leading to an increase in global mean surface temperature of 3.5 °C, 2 °C and 1.5 °C relative to pre-industrial levels. These scenarios present a broad spectrum of potential total greenhouse gas emissions by 2100, which are both feasible and in line with the trends in emissions growth observed over the past twenty years [15]. Together with the SSP pathway, the RCP significantly shapes the evolution of the electricity grid in the IAM scenario, both in capacity and technological innovation. Consequently, under the SSP2-RCP 1.9 scenario, there is an anticipated acceleration in the electrification of industrial and transport sectors, coupled with a marked advancement in sustainable power generation technologies. This progression directly impacts the carbon footprint of electricity, including that consumed by electrolysers for ammonia production, contingent upon the demands of the IEA scenario.

While the IAM SSP2-RCP 6.0, 2.6, and 1.9 do not perfectly match the assumptions behind the IEA's STEPS and SDS scenarios, a pairing is made and presented in Table 1.

**Table 1:** Alignment between IMAGE storylines and the IEA scenarios.

IMAGE			IEA	
scenario	radiative forcing (W/m^2^)	°C of warming (in 2100 vs. pre-industrial levels)	scenario	°C of warming (in 2100 vs. pre-industrial levels)
SSP2-RCP6.0	6.0	+3.5	–	–
SSP2-RCP2.6	2.6	+2.0	STEPS	+2.6
SSP2-RCP1.9	1.9	+1.5	SDS	+1.65

The IEA Roadmap does not provide an equivalent to SSP2-RCP6.0 (Base). However, as the Base scenario represents an absence of climate policy, the 2020 regional technology shares are assumed to remain constant in the Base (3.5 °C) scenario. The scenarios are referred to by the IMAGE degrees of warming in the rest of this paper (3.5 °C, 2.0 °C, 1.5 °C). It is essential to remember that models and scenarios are meant to offer insight into possible futures but are not predictions or plans. The IMAGE model and IEA scenarios contain underlying assumptions and specifications, which can be found in their documentation.

### 2.2 Life cycle inventories (Step 2)

#### 2.2.1 Ammonia production technologies

Ammonia currently accounts for 65% of the hydrogen demand in the industry [16]. Hence, the supply of low-carbon hydrogen is critical to decarbonising ammonia production. Hydrogen made from natural gas is classified as grey [17], while coal gasification produces black (from black coal) or brown (from lignite) hydrogen [18]. Using these colour classifications, current ammonia production is about 70% grey and 30% black.

If grey, black, or brown hydrogen is combined with carbon capture and storage (CCS) to prevent the process emissions of CO_2_ from entering the atmosphere, it is blue hydrogen [19]. Hydrogen from methane pyrolysis is turquoise hydrogen [20]. Despite the fossil fuel feedstock, scientists consider turquoise hydrogen a tool for a more sustainable hydrogen economy due to the solid carbon (not gaseous CO_2_) by-product [21]. Methane pyrolysis is not yet ready for industrial use and will require more development before commercialisation [22].

A key technology for the development of sustainable hydrogen is water electrolysis, which uses an electric current to split water into hydrogen and oxygen [23]. Alkaline electrolysis (AE) is mature and is the most common way of producing hydrogen electrochemically, followed by polymer electrolyte membrane electrolysis (PEM). Both technologies are commercially available and becoming more widespread, while solid oxide electrolysis (SOE) is developing [24].

Hydrogen produced from electrolysis can be classified as yellow if it comes from available grid electricity and green if the electricity comes from renewable sources [19]. While green hydrogen has the lowest *direct* GHG emissions, renewable energy will likely not be available at the scale needed for global hydrogen supply in the short term, making transition technologies attractive [21]. Electrolysis is responsible for only ∼0.03% of global hydrogen production due to the high cost and the electricity mix producing yellow hydrogen with a much greater emission intensity than grey hydrogen [16].

#### 2.2.2 Life cycle inventory development

The IEA Roadmap distinguishes the following production routes: Electrolysis, Pyrolysis, Coal, Coal with CCS, Gas, Gas with CCS, Oil, and Fossil with CCU (carbon capture and utilisation) [7]. Here, Fossil with CCU refers to CO_2_ capture for use in the industry (typically for urea), and CCS represents the compression, transport, and underground storage of emissions with a capture rate of 90% [7].

**Fig. 2:**
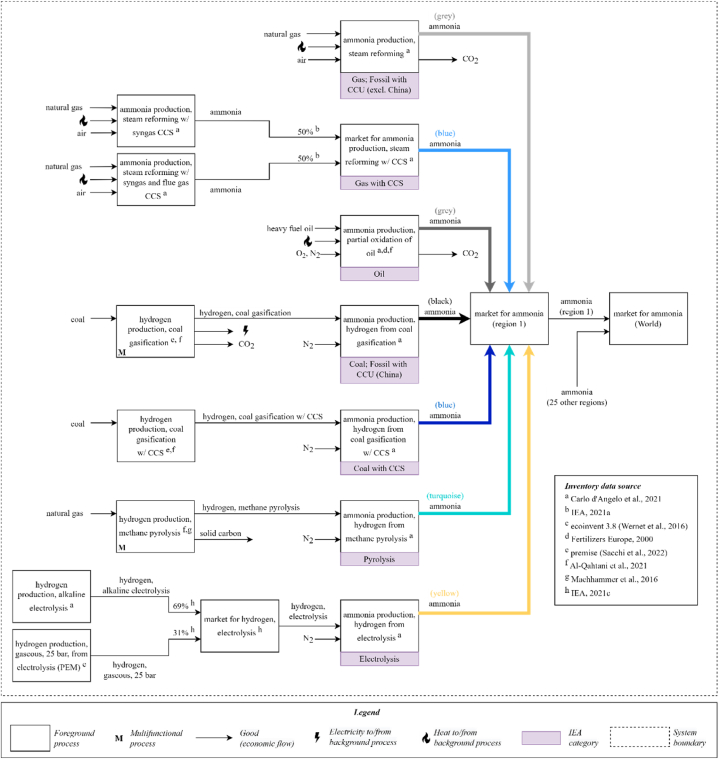
Key inventories in the new ammonia model and the corresponding IEA categories (purple boxes). Letters in superscript indicate the data sources for each process. Some input flows (such as electricity) are not shown, as they are common to all processes. (For interpretation of the references to colour in this figure legend, the reader is referred to the Web version of this article.)

The inventories in the ecoinvent database consist of gas- and coal-based ammonia production routes using outdated or non-transparent data sources. First, these were updated based on the work of Carlo d’Angelo et al. [25]. Second, additional inventories from literature were used to represent the other production routes: partial oxidation of oil [26,27], coal gasification (with CCS) [26] and methane pyrolysis [26,28]. [[25], [26], [27], [28]] Fig. 2 illustrates the modelled system. These life cycle inventories were all published in peer-reviewed journals recently (from 2016 to 2021), except for the some data for partial oxidation of oil, which was published in 2000 [27]. However, this technology has not developed in recent years and is being phased out of ammonia production worldwide.

Carbon dioxide captured for “use” typically goes to urea production in the ammonia industry. Since these emissions are eventually released during the fertiliser's use, the IEA “fossil with CCU” production quantities in this model are grouped as grey/black ammonia. This is not blue ammonia, as the CO_2_ will not be permanently stored.

Note that all ammonia production processes described use the Haber-Bosch process to convert hydrogen and nitrogen into ammonia. The nitrogen is taken directly from the air for the SMR route, while a designated nitrogen input (from cryogenic air separation) is used for the other routes. For SMR with and without CCS and partial oxidation of oil, the hydrogen production and Haber-Bosch processes are modelled in the same unit process. The hydrogen supply for the remaining four production routes is modelled separately.

Alkaline-based electrolysers supply 69% of the electrolytic hydrogen, while 31% comes from proton exchange membrane (PEM)-based electrolysers. This split reflects the current capacity of each electrolyser technology: 61% AE, 31% PEM, and 8% unspecified or SOE [29], with SOE excluded and the dominant technology (AE) assumed to cover the difference. The electrolysers are powered by the region-specific electricity grid, yielding yellow ammonia (not green). This modelling choice may differ from future observations, as there may be a shift to using a dedicated source of renewable electricity for hydrogen production. Therefore, this study's results can be considered a conservative estimate, as green ammonia could decarbonise the industry earlier than yellow ammonia. However, if low-carbon electricity sources are reserved for producing hydrogen, they become unavailable for other electricity-based systems.

### 2.3 Regionalisation (Step 3)

A “market” dataset in ecoinvent represents a consumption mix for a particular region [30]. Regional market datasets for ammonia are created. Supply shares of a given market for each production route vary over time and regions. Fig. 2 provides an example of a region (region 1) where the seven ammonia production routes supply the regional market with respective shares defined by the scenario. The IEA Roadmap provides the shares over time for eight IEA regions. After being mapped to the 26 IMAGE regions, these regional markets are linked to downstream ammonia users in the ecoinvent database. For example, the environmental impact of food products is influenced by a change in the ammonia market via the use of fertilisers on crops.

#### 2.3.1 Developments in ammonia production volumes

Time series expressed in megatonnes for each production route and region are built based on the STEPS and SDS scenarios of the IEA Roadmap for 2020 (actual), 2030, 2040, and 2050. Linear interpolation is used for the intervening years. The 2020 regional production volumes are provided for the top ammonia producers (China, Russia, Europe, USA, Middle East, and India). In addition to the volume changes, electrolysis efficiency improvements are applied for all three scenarios: 66% in 2020 and 76% in 2050 for both electrolyser technologies [11].

### 2.4 Integration of prospective data (Step 4)

This linking of the background and foreground systems, integrating ammonia and electricity scenarios, and regrouping into the IMAGE regional markets is performed using the Python library *premise* [31]. Prospective LCI databases are generated by *premise* by modifying the ecoinvent database to align with a given set of projections at each scenario and time step [14]. The prospective LCI databases are converted into a *superstructure* database and a corresponding *scenario difference file*, thus implementing the superstructure approach [32] using the open-source LCA software Activity Browser [33]. This facilitated calculating the life cycle environmental impacts of ammonia production across technologies, years, and climate scenarios. Provided access to open-source libraries *premise* and *Activity Browser*, the code and data necessary to reproduce Steps 1 to 4 are available in the scenario data repository (see Data availability section).

### 2.5 Life cycle impact assessment (Step 5)

The environmental impacts are expressed against several midpoint indicators provided by the set of methods called Environmental Footprint (EF) v3.0, as recommended by the European Commission [34]. Among the indicators in EF v3.0 is the impact category Climate Change, which is presented as Global Warming Potential (GWP) with a 100-year time horizon. Because the 2.0 °C and 1.5 °C IMAGE scenarios rely on using negative emissions technologies to reach climate targets (i.e., bioenergy with CCS (BECCS)), the uptake and release of biogenic CO_2_ are accounted for in the GWP calculation. Life cycle impact results (per kilogram ammonia produced) are generated for each production process, region, time step, and scenario. This research focused on greenhouse gas emissions, but other impact categories are reviewed.

### 2.6 Analysis of LCA results (Step 6)

The projected regional production totals could translate the per kg impacts to total regional and global effects. An in-depth review of the environmental impacts is performed. The influence of the ammonia scenarios on downstream food crops is also reviewed. The robustness of the model and results are also tested by applying it to a different integrated assessment model: REMIND. The key findings are summarised in Section 3: Results and Interpretation.

## 3 Results and interpretation

### 3.1 Global warming impacts of ammonia production

#### 3.1.1 Relative impacts

**Fig. 3:**
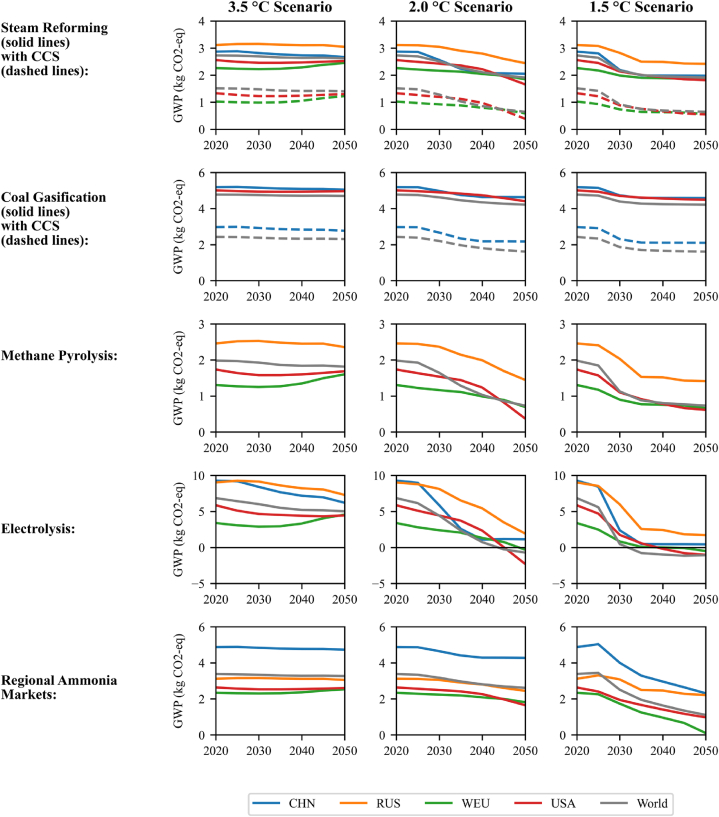
Global warming potential of 1 kg ammonia produced per technology, region, and scenario, as well as for the regional production mixes (markets). Negative values occur due to biogenic carbon sequestration (i.e., BECCS) in the background electricity scenarios.

The relative environmental impacts of each ammonia production technology are valuable results that can help guide policy development and investment decisions. In Fig. 3, climate change impacts (in kg CO_2_-eq per kg of ammonia) are compared for the modelled technologies. Note that a region is not displayed in a technology's subplots if the technology is not in its IEA scenario.

Partial oxidation of oil maintains a global warming potential of 3–4 kg CO_2_-eq per kg ammonia (lower only than coal gasification and fossil-based electrolysis ammonia). Representing less than 1.5% of global ammonia production in all scenarios, it is excluded from Fig. 3 and the discussion of critical technologies.

Initially, the technology with the lowest emissions is steam reforming with CCS (90% capture), but it is not used in all ammonia-producing regions. Coal gasification and early (fossil-fuelled) electrolysis create the most emissions. Coal-based ammonia is associated with high levels of GHG emissions because of the poor efficiency of the underlying hydrogen production process (∼9 kg of coal/kg H_2_ [35], or 51% efficiency based on the lower heating value, as opposed to 75% for the natural gas-based alternative [36]), combined with the low hydrogen-to-carbon ratio of coal (i.e., ∼2 kg of CO_2_ per kg of gasified coal are released in the atmosphere in the absence of capture), and upstream methane emissions (i.e., where close to 80% from the GHG emissions caused by coal supply are methane slips during coal extraction [30]). Vast reserves of coal (and available infrastructure to distribute it) have incentivized the production of coal-based hydrogen (and ammonia) in China, driving production costs down to half that of electrolysis-based hydrogen [37], making it challenging for the country to transition away from this production route. Yet, better alternatives are envisioned in the future in the sustainable scenario. Carbon capture and storage offer a possible solution to mitigate GHG emissions caused by coal gasification. However, it requires energy to separate CO_2_, and CCS depends on transportation and accessible sites for storage [11]. Electrolysis, on the other hand, becomes the lowest emitter in later years of the 1.5 °C scenario, making it a key technology.

The emerging technology methane pyrolysis (turquoise ammonia) only plays a minor role in the sustainable scenario (less than 3.5% of production), but it has been included for comparison purposes. Methane pyrolysis has the potential to reduce CO_2_ emissions from ammonia significantly but will need to be developed further before it is viable [11]. This also means its life cycle inventory could change drastically when the technology has scaled up. The input data and results are considered less reliable than the more established technologies. Modelling decisions, such as the economic allocation of environmental burden to the solid carbon co-product, also affect the outcome. More research should be done into methane pyrolysis before conclusions are drawn about this technology. In addition, the continued reliance on non-renewable resources such as natural gas may be a reason to avoid investing in turquoise ammonia over green ammonia.

In 2020, the average global impact across the regions is 3.4 kg CO_2_-eq/kg ammonia. By 2050, this per-kilogram impact would decrease by 4%, 23%, and 67% in the 3.5 °C, 2.0 °C, and 1.5 °C scenarios, respectively. In the most sustainable scenario, the 2050 average global impact is 1.1 kg CO_2_-eq/kg ammonia.

The Chinese technology mix has the highest global warming impacts due to the heavy use of coal gasification. It substantially improves under the 1.5 °C scenario but remains the worst emitter. However, it becomes almost comparable to Russia in 2050, as Russia has no steep decarbonisation in the IEA ammonia roadmap or IMAGE electricity model. The other regions are modelled with much more substantial emissions reductions. Though Western Europe gets very close, none of the areas achieves net-zero GHG emissions with respect to ammonia under these scenario conditions.

#### 3.1.2 Global impacts

**Fig. 4:**
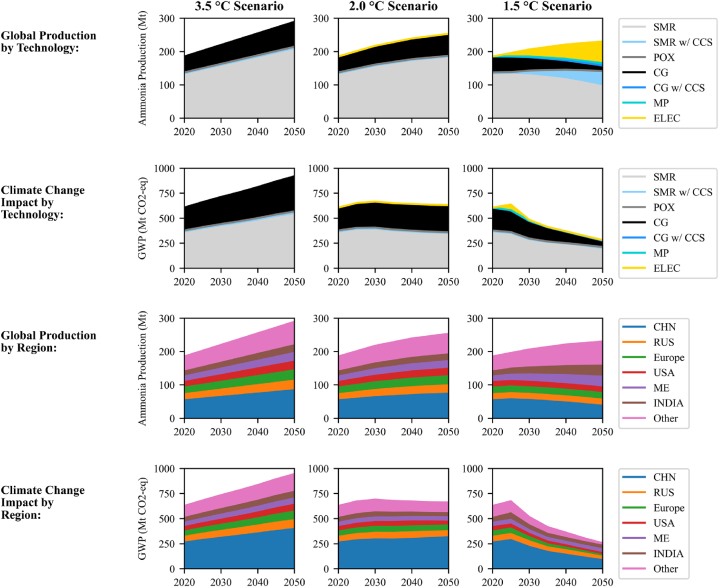
Overall ammonia industry production volume and climate change impact (GWP) (including relative contributions of different technologies and the top producers).

The relative technology/region impacts are used to shed light on the overall ammonia industry. Fig. 4 provides an overview of the modelled production volumes and the climate change impact of global ammonia production. Production data stems from the IEA Roadmap converted to IMAGE regional groups.

Under the scenarios and assumptions used in this analysis, the ammonia industry will not be fully decarbonised by 2050. In the most ambitious of the three storylines, 1.5 °C, ammonia production will still be responsible for 255 Mt CO_2_-eq emissions in 2050. This is double the value given in the IEA study because LCA captures more upstream and downstream impacts and infrastructure, not only direct emissions. The underlying electricity modelling may also be different in the IEA roadmap. However, this illustrates the value of firm climate policy. Left unchecked (as in the 3.5 °C scenario), the emissions could reach almost 950 Mt CO_2_-eq in 2050 (over 3.5 times greater than the 1.5 °C scenario). The 2.0 °C scenario has 660 Mt CO_2_-eq in 2050 (over 2.5 times greater than 1.5 °C).

SMR and coal gasification (CG) dominate in the 3.5 °C and 2.0 °C scenarios. In the 1.5 °C scenario, however, there is a decrease in fossil fuel technologies and an increase in alternatives: SMR with CCS and electrolysis. Electrolysis is modelled as yellow ammonia, but much of it could be green ammonia, discussed further in section 4.2.

The impact of electricity plays a minor role in the first two scenarios since there is little ammonia production via electrolysis. For 1.5 °C, in contrast, the background electricity scenarios are vital to the results. Reducing the impact of ammonia cannot occur without a technology shift to electrolysis combined with an electricity system transition to renewable energy.

To limit global warming to 1.5 °C (67% likelihood), the remaining carbon budget (from the start of 2020) is 400 gigatonnes (Gt or billion tonnes) of CO_2_ [38]. The cumulative impact of ammonia production from 2020 through 2050 will use 6%, 5%, and 4% of this carbon budget for 3.5 °C, 2.0 °C, and 1.5 °C, respectively (24, 21, and 15 Gt).

The main impact comes from the continued use of coal gasification and steam reforming. If green ammonia displaces a larger market share and stricter policy measures are implemented, this number could be lowered.

One reason fossil technologies still play a significant role in 2050 is urea production. While some fossil ammonia plants may be retrofitted with CCS, this major fertiliser requires a convenient source of CO_2_, which wouldn't be available from electrolysis, methane pyrolysis, or the CCS technologies. This model used the same handling of steam reforming CO_2_ emissions as ecoinvent 3.8, where all CO_2_ produced is an emission, whether or not it is used downstream for urea.

This will remain necessary until demand decreases or there is a shift to alternative fertilisers. More than half of the ammonia currently produced globally is used for urea, so fossil ammonia cannot be easily substituted with green ammonia. However, biomass gasification is a potential alternative [39]. The IEA's sustainable development scenario (matched with 1.5 °C) shows a 28% reduction in urea fertiliser use by 2050, shifting to ammonium nitrate and calcium ammonium nitrate [7]. Also, organic fertilisers (e.g., compost, manure [40]) could be further promoted to reduce the reliance on urea. Finally, if the transition to urea alternatives proves too challenging, it could be synthesized by co-reducing NO_3_^−^ and non-fossil CO_2_ [41], compensating the CO_2_ emitted when urea undergoes hydrolysis and nitrification.

To achieve complete decarbonisation of the ammonia industry (which is not accomplished in these scenarios), a significant expansion of green ammonia on a large scale (dependent on political determination and rapid implementation of renewable electricity) and replacements for urea would be necessary.

The IRENA report anticipates that all new ammonia production capacity will be renewable after 2025 and cost-competitive with blue ammonia after 2030 [39]. Despite this promising outlook, many conventional ammonia plants are unlikely to be decommissioned early. Chinese plants, in particular (average age 12 years), are not yet close to their usual “lifetime” (50 years) [7].

#### 3.1.3 Regional impacts

The reader can consult the scenario data repository (see Data availability section) to obtain detailed impact results by supply technology and region for all years and scenarios.

### 3.2 Other impact categories

While this report focuses on greenhouse gas emissions, an interesting feature of LCA is identifying “burden shifting” from one impact category to another. It's essential to consider other environmental impacts and trade-offs when making policy, investment, and design decisions. Impact categories other than climate change are less well-defined and reviewed. As such, these results should be considered cautiously.

**Fig. 5:**
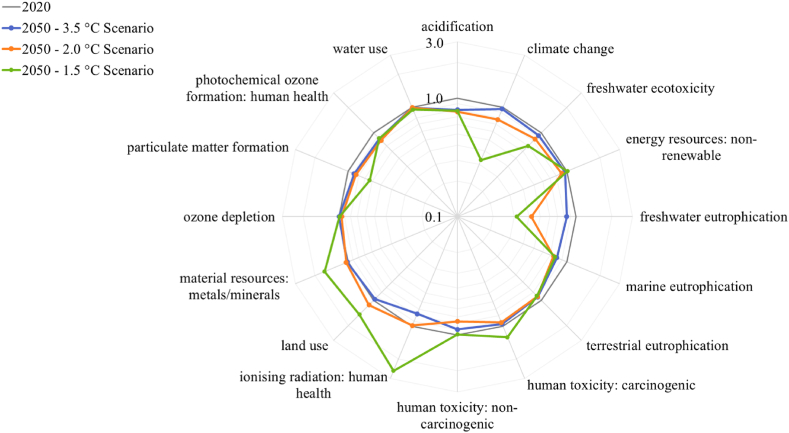
Global average impact per kg ammonia in 2050 for all scenarios (multiple impact categories) relative to initial (2020) values (2020 has a value of 1.0 for all categories). The axis uses a logarithmic scale with base 10.

Fig. 5 shows the percent difference between the total global impact of multiple impact categories in the 3.5 °C, 2.0 °C, and 1.5 °C scenarios in 2050 compared to the baseline in 2020.

Some categories (including climate change) decrease the most in the 1.5 °C scenario, while others rise (e.g., land use, metal depletion, ionising radiation, and human toxicity). These are tied in general to the energy transition. In this study, a look into regions such as Western Europe showed that the increase in the impact of electrolysis's land use in the later years of the 2.0 °C and 1.5 °C scenarios is due to the extensive use of BECCS to achieve negative emissions. Metal demand is tied to the development of renewable energy generation in an increasingly decentralised power system (as is metal mining to human toxicity), and ionising radiation results from uranium mining for nuclear power.

### 3.3 Impacts of downstream products

The use of ammonia in fertilisers has wide-ranging environmental effects that impact essential food products. In 2018, four major crops (sugar cane, maize, rice, wheat) accounted for half of the world's primary crop production, with respective quantities of 1.9 billion tonnes, 1.1 billion tonnes, 0.8 billion tonnes, and 0.7 billion tonnes [42]. When considering the fossil GWP – excluding biomass CO_2_ uptake – the environmental impact of these crops diminishes in the 1.5 °C scenario due to improved ammonia and electricity production practices. By examining representative regional crop production processes (Brazilian sugar cane, USA maize, Chinese rice, and Indian wheat), the GHG emissions per kilogram decreased by 10%, 18%, 26%, and 20%, respectively, between 2020 and 2050. These reductions are significant given the substantial quantities of these crops consumed annually.

### 3.4 Comparison to a REMIND-based model

A comparison is made between the IAM REMIND model [43] and the IMAGE model. The REMIND model used different radiative forcing scenarios (2.0 °C aligns with REMIND SSP2-PkBudg1100 and 1.5 °C with PkBudg900) and had lower geographical resolution (12 regions compared to IMAGE's 26). However, the overall climate change impact results are similar. The GWP of 1 kg ammonia from REMIND differed by less than 10% from IMAGE in each corresponding year and scenario for the World market. One notable difference is that REMIND relied more on faster renewable energy development and less on BECCS, indicating different underlying technologies for decarbonisation. This alternative approach reduces the burden on land use compared to using BECCS.

## 4 Discussion

### 4.1 Application of scenarios

The IEA Roadmap, guiding this prospective LCA, outlines three scenarios for ammonia's agricultural and industrial use, ranging from current trends to alignment with the Paris Agreement. These scenarios consider economic and scalability factors, environmental and social impacts, and urea and ammonia fertiliser demand shifts. The article highlights vital decarbonisation challenges, including the higher costs of emerging ammonia production methods, the crucial role of policy for market creation, and the necessity for both technological and structural changes. Current limitations include the commercial readiness of technologies like carbon storage and hydrogen production from renewable sources. It also stresses the significance of non-CO_2_ emissions, such as nitrous oxides and methane, and the environmental impact of fertilisers' over-application versus their role in preventing additional agricultural land use. This study focuses on LCA outcomes rather than the feasibility of these scenarios, using them as analytical tools rather than predictive models. For detailed insights, the Roadmap document itself should be consulted.

### 4.2 The importance of prospective LCA

LCA practitioners performing prospective studies on ammonia or ammonia-containing products could overestimate the climate change impact by up to 70% for 2050 by relying on ecoinvent 3.8 “present day” data. Evaluating future technologies based on past data can lead to several missteps, such as investing in the wrong technology (ending with sunk costs) or missing the potential of a future “green” technology because it is not the most sustainable option today. While results based on models of the future must always be taken with caution, they can provide valuable insight. To illustrate the value of future background scenarios in pLCA, a comparison is made between the average impact of ammonia from the current inventories in ecoinvent and the scenario inventories from this study. For 2020, the result from ecoinvent 3.8 is only 7% greater than the updated inventories. However, it would differ if the current ecoinvent data are used for 2050. This model shows that the climate change impact could be 11%, 28%, and 70% lower than the current ecoinvent value (in the three increasingly sustainable scenarios). This can significantly impact the results of a prospective LCA with electricity or ammonia used in the background.

These ammonia scenarios are available online^a^ for IMAGE and REMIND models and are intended to be used on top of the corresponding electricity scenarios. This will help to improve the quality of pLCA and reduce the temporal mismatch in pLCA studies between foreground and background data.

This study included background scenarios for electricity and ammonia. *premise* has also been used to incorporate the developments of other sectors such as cement, steel, and transport [14]. Similar methods to build custom scenarios in pLCA were used for copper, nickel, zinc, lead [44] and cobalt [45].

The life cycle assessment approach also considers emissions from the upstream infrastructure and background processes and the use of fertiliser, which also releases emissions. This, and the choice of background electricity models, resulted in double the total CO_2_ emissions in 2050 for this study compared to the IEA roadmap. This highlights the importance of a life cycle approach to measure true decarbonisation.

### 4.3 The role of electrolysis and green ammonia

A key observation is that the choice of scenario significantly impacts ammonia production via hydrogen from electrolysis. In the 3.5 °C scenario, electrolysis remained a high emitter over the entire period. However, for many regions, electrolysis ammonia became the technology with the lowest global warming potential by ∼2045 in the 2.0 °C scenario and ∼2035 in the 1.5 °C scenario due to the decarbonisation of their electricity grid.

This assumes that companies will use grid electricity for ammonia production. However, companies may also purchase electricity from renewable sources like wind or solar power. Ammonia produced from renewable electricity is the option with the lowest GHG emissions. In China, for example, the results are 0.3 kg CO_2_-eq. per kg of ammonia produced via electrolysis with onshore wind power, compared to close to 10 kg CO_2_-eq. with Chinese grid electricity. However, the results for green ammonia depend on the source of renewable electricity, the production location, the electrolyser technology used, and other factors.

In conclusion, yellow ammonia is the best option regarding GHG emissions, providing grid-based electricity with a low carbon intensity. Electrolysis-based ammonia is undoubtedly essential in reducing GHG emissions: producing 500 megatonnes of ammonia in 2050 using anything other than electrolysis or SMR with CCS would significantly increase the overall GHG emissions. While the study's observations are based on various assumptions and simplifications, they provided valuable insights into the potential impact of ammonia production via hydrogen from electrolysis under different scenarios. The decarbonisation of the ammonia industry is very strongly tied to the decarbonisation of the electricity sector. The speed at which renewable energy grows will determine if the outcome is closer to the 1.5 °C scenario or 3.5 °C scenario by 2050.

However, electrolysis also requires resources beyond electricity itself. This study assumed a static ratio of alkaline and PEM electrolysis, improving efficiencies from 66% to 76% [11]. A sensitivity analysis showed that adjusting the future division between PEM and AE did not significantly change the climate change results. However, a global material flow analysis may be more appropriate for assessing differences between electrolysis technologies regarding raw material criticality. The scarcity of iridium, which may limit the growth of PEM technology unless there is a sharp decrease in iridium loading and an increase in recycling rates [46], is not visible in these LCA results. The future of electrolysis is uncertain, and newer technologies, such as solid oxide electrolysis, are emerging.

Finally, it should be noted that scaling electrolysis capacity to produce the required amount of yellow ammonia may be a challenge: Odenweller et al. [47] demonstrate that even if electrolysers experienced the same exponential growth as solar and wind power did, green hydrogen may barely represent 1% of the global final energy demand by 2035, further noting that the installed electrolysis capacity would need to grow 6000–8000-fold compared to today to align with the requirements of the 1.5 °C scenario.

### 4.4 The impact of natural gas production

The global warming potential of ammonia production by steam methane reforming varies by region, with emissions due to background electricity production, transportation, coal mining, natural gas extraction/processing, and other factors playing a role. SMR from Russia had the highest life cycle emissions due to natural gas venting or flaring, as well as other processes such as heat and power co-generation. In contrast, WEU had the lowest emissions on average.

Methane, the main component of natural gas, is also a significant contributor to global warming, and methane leaks during production and transport are difficult to measure and often underestimated [48,49]. Energy sector methane emissions are approximately 70% higher than reported by national governments, according to the IEA [50]. While using natural gas through SMR with CCS has been presented as a green alternative to coal or other fossil fuels, the expansion of natural gas infrastructure is slowing the transition to renewable energy. Conventional natural and shale gas have more significant greenhouse gas footprints than coal or oil on a 20-year scale. Methane leaks vary in severity by region, with some of the worst areas identified as Turkmenistan, Russia, and the United States [51]. Harmful gas emissions are inevitable, even with the implementation of CCS technologies. For instance, in our analysis, using a time horizon of 100 years, approximately 5 kg of CO_2_-eq. emissions are produced per kg H_2_ generated via SMR of European natural gas with CCS, of which ∼1 kg is attributed to methane leaks along the distribution pipelines – and up to 2 kg CO_2_-eq. or 30% when using Russian natural gas.

Given these issues, the relative impacts of the technologies in this study should be considered critically, with the awareness that turquoise and blue ammonia still risk large amounts of fugitive emissions in the upstream processes. While they have relatively low climate change impacts compared to grey ammonia, they rely on the continued use of natural gas and efforts to minimize methane leaks. They are, therefore, not silver bullet solutions.

### 4.5 Burden shifting to other impact categories

Section 3.2 highlighted the importance of considering trade-offs in decision-making processes, as scenarios that improve one impact category may shift the environmental burden to another. Reliance on BECCS as a solution for reducing greenhouse gas emissions has raised concerns over land-use competition and other environmental impacts. Indeed, the land use impacts are highest in the 1.5 °C scenario. It's crucial to view these solutions as part of a broader environmental strategy that includes sharp global prevention of industrial emissions [52].

An in-depth investigation into the trade-offs associated with the increased electricity and biomass demand required for the future of ammonia is needed. A regional analysis is necessary to determine the best paths to a secure, sustainable future and food (fertiliser) system while minimising the negative environmental impact. Rosa and Gabrielli [53] have explored the trade-offs associated with transitioning to emissions-free methods of ammonia production, considering food security, energy, and environmental implications such as land and water use.

Water use is another point of debate when discussing electrolysis ammonia. However, Beswick et al. [54] have shown that renewable electricity used for hydrogen production in the ammonia industry has a lower water demand than fossil energy. This LCA study agreed with that finding, with ammonia from SMR having at least 1.5 times higher water use impacts than electrolysis hydrogen.

Besides land use, material depletion of metals and minerals also rose in the sustainable scenario, driven mainly by copper mining. Copper demand is linked to the growth of renewable energy systems due to their high electrical conductivity and low substitution potential [55]. Copper smelting contributes to carcinogenic and non-carcinogenic human toxicity impacts in the 1.5 °C scenario. In addition, ionising radiation increases by over 200% in the 1.5 °C scenario due to the growth of nuclear energy in the IMAGE model.

### 4.6 Limitations and future research

This study aimed to improve future background coverage of ammonia in the chemical industry, but limitations remained due to simplifications and assumptions. The background IMAGE electricity storylines and IEA ammonia scenarios did not align perfectly, affecting comparability since different sources' scenarios are mixed. Nevertheless, this scenario alignment yielded more accurate results than a prospective LCA, which does not cover developments in the background system.

While this research focused on ammonia in the chemical industry, there is potential for significant growth in ammonia as a fuel, energy carrier, or storage medium. Ammonia produces no CO_2_ emissions when combusted, has a high energy density, and is easier to transport and store than pure hydrogen [7]. These emerging uses of ammonia are excluded from the main scope of the IEA Roadmap, but they should be included when assessing the future capacity of the ammonia industry. If maritime fuel and power generation are added, the SDS 2050 ammonia demand could almost double from 230 Mt for existing agricultural/industrial uses to over 400 Mt [7]. However, this ammonia application is still developing, requiring high temperatures to burn and producing dangerous nitrogen oxides during combustion [56].

Some forecasts project even more significant growth in emerging ammonia sectors. The IRENA 1.5 °C scenario expects almost 200 Mt and 130 Mt of new (renewable) ammonia demand by 2050 due to the maritime industry and the trade of ammonia as a hydrogen carrier, respectively [39]. Their 2050 projection of ammonia demand for existing applications is higher than the IEA's. It results in a 2050 total ammonia demand of 688 Mt and 566 Mt, which would come from renewable energy sources. The global impact will depend on the overall mix of production technologies and the type of electricity used for electrolysis ammonia. However, to supply green hydrogen (or ammonia) to decarbonise the chemical industry, other hard-to-abate sectors, and emerging applications such as fuels, there must be a vast and rapid increase in renewable energy installations to meet the demand [57].

Moving to electrolyser-based hydrogen will likely change ammonia plant sizes since these could be smaller operations [58]. This may result in decentralised production and different logistics strategies or business plans worth investigating in future industry models. The average transport distance given to all new ammonia markets is also based on ecoinvent 3.8. Therefore, they can be over- or under-estimated and are not regionally differentiated. Transportation may become more significant in the overall impact of ammonia in the future, especially if plants are more distributed and sources of renewable energy need to be nearby.

Off-grid green ammonia configurations are not considered. However, if hydrogen and ammonia producers can overcome intermittency issues, they may move towards an off-grid electricity supply, choosing to power electrolysers with on-site renewable energy. This global model did not include that level of site-specific complexity. Considering case-specific prospective LCAs to design new ammonia installations could be valuable.

## 5 Conclusions and recommendations

This research aimed to investigate the future of ammonia production and its impact on the climate. The study used the IEA Ammonia Roadmap and IMAGE electricity scenarios to produce an extensive prospective LCI database for scenario LCAs across three storylines from 2020 to 2050. The global scope and application of scenarios allowed for extended exploration of the ammonia industry's potential future pathways and related environmental impacts. The development of these inventories and integration with the *premise* tool allow for their use by other LCA practitioners in their work.

It is found that the most polluting ammonia production route is via coal gasification. At the same time, the option with the lowest climate change impact is electrolysis from renewable electricity sources, followed by steam reforming with CCS. Yellow ammonia (electrolysis with grid electricity) is initially quite polluting but becomes viable later in the 1.5 °C scenario when the electricity mix is cleaner. It is emphasised that the ammonia industry is intertwined with other sectors and cannot be decarbonised without low-carbon electricity.

Electrolysis with renewable electricity is the best way to decarbonise the industry, but critical material scarcity could limit its use. Reducing urea demand is also essential for reducing reliance on SMR with CCU, and a swift uptake of electrolysis with designated renewables must be the focus. In addition, background processes such as extraction and production of natural gas have many hidden impacts and highlight the problems with relying on fossil fuel feedstocks despite carbon capture technology and emerging technologies such as methane pyrolysis.

Even in the 1.5 °C scenario, the ammonia industry is unlikely to achieve complete decarbonisation by 2050, anticipating residual emissions of 255 Mt CO_2_-equivalent, caused mainly by natural gas-based ammonia still supplying 40% of the demand globally. However, the other situations are 2.5 or 3.5 times worse (2.0 °C or 3.5 °C scenario respectively). From 2020 to 2050, the ammonia sector's cumulative emissions are estimated at 24, 21, and 15 gigatonnes of CO_2_-equivalent for the 3.5 °C, 2.0 °C, and 1.5 °C scenarios respectively, surpassing the IEA's forecasts due to the comprehensive life cycle analysis employed. This underscores the potential for green ammonia, derived through electrolysis, to assume a more critical role should its production scale sufficiently in that timeframe [47]. Additionally, it highlights the urgency of curbing demand to reduce reliance on ammonia made from natural gas. Given that only 32% of current crop production (by calorie) directly supports human nutrition [59], with the remainder allocated to direct animal feed (23%), various other purposes (40%, inclusive of indirect animal feed), and covering seed and waste (5%), reducing meat consumption emerges as a strategic approach to decrease ammonia demand, potentially to the extent of fully obviating the need for its fossil-based counterparts.

Crops and processed food products that use ammonia are also going to evolve. Based on their background electricity and ammonia fertiliser consumption, many food products reduce CO_2_ impacts (10–26% in the 1.5 °C scenario).

The 1.5 °C storyline requires a sufficiently high carbon price to drive the growth of renewable ammonia alternatives [7]. The current lack of emissions regulations restricts the development of green ammonia since it is not yet cost-competitive, and strong policies are needed to accelerate it [39]. Immediate efforts must be made to increase low-carbon electricity production and reduce ammonia fertiliser demand.

The discussion highlighted the importance of keeping the LCI inventories current, particularly for emerging technologies such as methane pyrolysis, and of further research into critical material scarcity and land competition. The method used in this research can also be applied to other products, such as methanol and fuels, thereby increasing the future background scenario coverage for major industrial polluters.

The prospective LCA considered ammonia as a chemical and did not include the potential growth of ammonia used as a fuel or energy carrier. The excess demand for these applications would require an even more significant expansion of renewable energy. Future research should consider this and the potential decentralised nature of future ammonia production.

## Data availability

The scenarios discussed in this paper can be found in the repository https://github.com/premise-community-scenarios/ammonia-prospective-scenarios. This data, to be used in the open-source prospective life cycle assessment framework *premise*, allows the reproduction of the results presented in this study. The results presented in this study are included in this repository, including time series for regional impacts.

## CRediT authorship contribution statement

**Johanna Boyce:** Writing – review & editing, Writing – original draft, Visualization, Software, Project administration, Methodology, Investigation, Formal analysis, Data curation, Conceptualization. **Romain Sacchi:** Writing – review & editing, Writing – original draft, Visualization, Validation, Supervision, Software, Methodology, Conceptualization. **Earl Goetheer:** Validation, Supervision, Conceptualization. **Bernhard Steubing:** Writing – review & editing, Writing – original draft, Supervision, Methodology, Conceptualization.

## Declaration of competing interest

The authors declare that they have no known competing financial interests or personal relationships that could have appeared to influence the work reported in this paper.

## Footnotes

a https://github.com/premise-community-scenarios/ammonia-prospective-scenarios.
